# Near-Field to Far-Field RCS Prediction on Arbitrary Scanning Surfaces Based on Spherical Wave Expansion

**DOI:** 10.3390/s20247199

**Published:** 2020-12-16

**Authors:** Woobin Kim, Hyeong-Rae Im, Yeong-Hoon Noh, Ic-Pyo Hong, Hyun-Sung Tae, Jeong-Kyu Kim, Jong-Gwan Yook

**Affiliations:** 1 Department of Electrical and Electronic Engineering, Yonsei University, Seoul 03722, Korea; woobink0203@yonsei.ac.kr (W.K.); ihr3021@yonsei.ac.kr (H.-R.I.); yh.noh@yonsei.ac.kr (Y.-H.N.); 2 Department of Information and Communication Engineering, Kongju National University, Cheonan 31080, Korea; iphong@kongju.ac.kr; 3 Aerospace Technology Research Institute, Agency for Defense and Development (ADD), Seosan, Chungnam 32024, Korea; hyunsung2000@add.re.kr (H.-S.T.); jeong806@add.re.kr (J.-K.K.)

**Keywords:** radar cross section (RCS) measurement, near-field to far-field transformation (NFFFT), spherical wave expansion (SWE)

## Abstract

Near-field to far-field transformation (NFFFT) is a frequently-used method in antenna and radar cross section (RCS) measurements for various applications. For weapon systems, most measurements are captured in the near-field area in an anechoic chamber, considering the security requirements for the design process and high spatial costs of far-field measurements. As the theoretical RCS value is the power ratio of the scattered wave to the incident wave in the far-field region, a scattered wave measured in the near-field region needs to be converted into field values in the far-field region. Therefore, this paper proposes a near-field to far-field transformation algorithm based on spherical wave expansion for application in near-field RCS measurement systems. If the distance and angular coordinates of each measurement point are known, the spherical wave functions in an orthogonal relationship can be calculated. If each weight is assumed to be unknown, a system of linear equations as numerous as the number of samples measured in the near electric field can be generated. In this system of linear equations, each weight value can be calculated using the iterative least squares QR-factorization method. Based on this theory, the validity of the proposed NFFFT is verified for several scatterer types, frequencies and measurement distances.

## 1. Introduction

In many cases, radar cross section (RCS) measurements for an object under test (OUT) or the radiation patterns of an antenna under test (AUT) are captured in the near-field region inside an anechoic chamber or at an outdoor site. The theoretical RCS value is the power ratio of a scattered wave measured at infinity to an incident plane wave. The far-field region is proportional to the electrical size of the OUT compared to the wavelength. Specifically, it is calculated as $2d^{2}/\lambda$, where *d* is the linear size of the OUT and λ is the wavelength. This implies that, as the target expands or the frequency increases, the far-field range criterion will become excessively large for practical measurements. Therefore, in many cases, a specially designed reflector is placed between the feeder and OUT to induce far-field conditions [1]. Another approach is the use of a near-field to far-field transformation (NFFFT) technique in a non-ideal measurement environment. Traditionally, field-transformation algorithms have focused on the processing of field values measured over canonical surfaces (e.g., planes [2], cylinders [3] and spheres [4]) using corresponding modal expansion techniques. To minimize measurement time, studies on non-uniform near-field sampling grids have been conducted continuously for each type of scanning surface [5,6,7]. In particular, several approaches have been presented for the arbitrary-probe NFFFT of spherical near-field measurements. These approaches include angular scanning [8] and probe modal content tuning [9,10,11,12]. However, modal expansion requires every point for measuring a near-field to exist on a surface with a single coordinate system in order to maintain orthogonality. Additionally, the far-field conversion process is unintuitive and complicated, because calculating the integral equation corresponding to each coordinate system is required for the NFFFT process. When measuring within a specific bandwidth rather than at a single frequency, the computational complexity increases significantly because the solution for the new integral equation must be calculated according to the wavenumber corresponding to the sampling frequency within the bandwidth. Additionally, gaps between measurement samples must exist on the canonical surface, which impairs the flexibility of near-field measurements. In a real measurement system, the flexibility of near-field sampling is crucial to avoid limiting the shape of the OUT or the measurement frequency.

**Figure 1:**
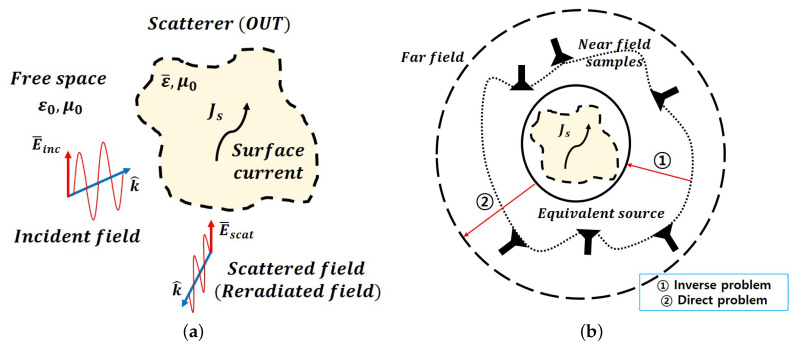
Schematic view of electromagnetic scattering and the process of spherical wave expansion–near-field to far-field transformation (SWE-NFFFT). (**a**) The geometry of electromagnetic scattering. (**b**) Illustration of SWE-NFFFT.

To overcome the limitations discussed above, an NFFFT algorithm that can flexibly convert an arbitrary near-field scanning surface measurement value into a far-field RCS within various frequency bands is required. In this paper, a far-field RCS prediction method based on the spherical wave expansion (SWE) method that adapts modal expansion techniques away from canonical surfaces is proposed. SWE-NFFFT has mainly been used to predict far-field radiation patterns based on near-field measurements of AUTs [13,14,15,16,17,18,19,20,21]. First, the point at which an AUT’s radiation field is located is assumed to be the origin of the coordinate system and an arbitrary near-field measurement point can be expressed in spherical coordinates. The electric field at a given location can be expressed as a weighted sum of spherical waves that are orthogonal to each other. Assuming that the weight multiplied by each spherical wave is unknown, a system of linear equations as numerous as the number of samples measured in the near electric field can be generated. The unknown weights can be calculated from the linear system of equations generated in this manner. This process is called an inverse problem. Next, the RCS defined in the far-field domain can be predicted using the calculated weights. This process is called a direct problem, and all processes in the inverse problem and direct problem can be considered as the basic principles of NFFFT, as illustrated in Figure 1.

The method described above can be applied to the RCS of an OUT in its current form. Regarding the process of generating scattered waves, when an electric field is incident on an OUT, a surface current density is induced on the OUT surface by the incident waves. This induced surface current density acts as a source and generates another electric field, which is regarded as a scattered wave reflected by the OUT. Therefore, an OUT on which an incident wave generates a surface current density can be modeled as an equivalent current source, such as an AUT emission field.

In this study, an NFFFT algorithm was developed based on the SWE theory to verify the validity of far-field RCS prediction for an arbitr-ry near-field scanning surface. First, NFFFT RCS results for two canonical surfaces (spherical and planar) scans were compared and analyzed using a NASA almond type OUT for which reliable reference measurement data were available. Next, the validity of SWE-based NFFFT for arbitrary scanning surfaces was confirmed based on the extracted far-field RCS results from scanning near-field samples for actual missile type OUTs with a larger electrical size.

## 2. Theory

### 2.1. Radar Cross Section

An RCS is utilized as a measure of the reflective strength of a target and is defined as 4π times the ratio of the power per unit solid angle scattered in a specified direction to the power per unit area in a plane wave incident on the scatterer in a specified direction [22]. Specifically, it is defined as the limit value of the distance between the scatterer and the point at which the scattered power is measured at infinity, as follows:

(1) $$\sigma =\lim_{r\to \infty }4\pi r^{2}\frac{|E_{scat}{|}^{2}}{|E_{inc}{|}^{2}}=\lim_{r\to \infty }4\pi r^{2}\frac{P_{scat}}{P_{inc}}\;\left[m^{2}\right]$$

(2) $$\sigma_{dBsm}=10\log_{10}\left(\frac{\sigma }{\sigma_{ref}}\right)=10\log_{10}\left(\frac{\sigma }{1}\right)\;\left[dBsm\right]$$

where *r* is the distance from the OUT to the measurement point, $E_{scat}$ is the scattered electric field and $E_{inc}$ is the incident electric field at the target. The unit for an RCS is square meters. Because objects for which RCSs are measured have various sizes, the logarithm power scale is often adopted, and 1 $m^{2}$ is used as a reference value, as shown in Equation (2).

**Figure 2:**
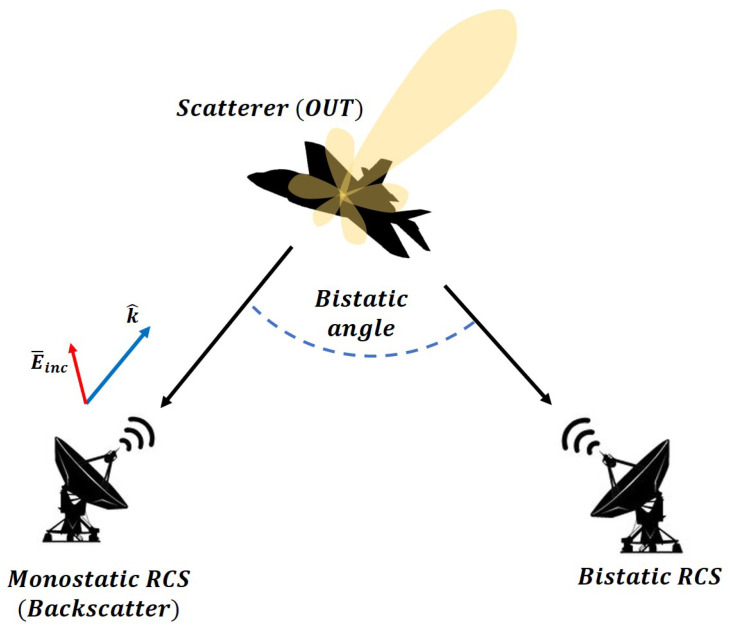
Illustration of monostatic and bistatic radar cross sections (RCSs).

As shown in Figure 2, depending on the direction in which a scattered wave is measured, the RCS can be categorized as monostatic or bistatic. If scattering is observed in the *k* vector direction, which is the opposite direction of the incident wave, then it is defined as monostatic RCS, while all other cases are defined as bistatic RCS.

Based on the definition above, the RCS measurement of an OUT can be performed using two methods: outdoor and indoor. According to the theoretical definition of an RCS, the measurement environment that is more suitable for measuring scattered waves at infinite distances is outdoors. However, it is necessary to consider the effects of various types of clutter that can affect RCS measurement values (e.g., weather conditions such as rain or snow or wide-open flat spaces). In most cases, indoor measurements can provide cleaner measurement data with reduced clutter, even though the measurement space is limited in size. In the near-field region, the shape of an electromagnetic wave is closer to the shape of a spherical wave than a plane wave. Therefore, if the power ratio is calculated or measured from a scattered wave in the near-field region, the RCS values will differ from the true values. Therefore, it is necessary to predict a far-field RCS in which a plane wave is reconstructed by applying a post-processing technique to the electric field value measured in the near-field region. The following subsection details the SWE-NFFFT theory for arbitrary scanning surfaces, which is not limited to the canonical scanning surfaces, unlike the conventional NFFFT algorithm.

### 2.2. Near-Field to Far-Field RCS Prediction Based on SWE

As shown in Figure 1, the scattered wave formed by an OUT can be derived from an equivalent surface current density by utilizing Love’s equivalence principle [23]. Specifically, the scattered wave formed by an OUT can be expressed as a weighted sum of spherical wave functions that are orthogonal to each other at an arbitrary measurement point, which matches the form of the following spherical wave expansion:

(3) $$\vec{E}\left(r,\theta ,\phi \right)=\sum_{s=1}^{2}\sum_{n=1}^{N}\sum_{m=-n}^{n}Q_{smn}{\vec{F}}_{smn}^{\left(3\right)}$$

(4) $$N=\lceil ka+6{\left(ka\right)}^{\frac{1}{3}}\rceil$$

where s=1,2 represent two orthogonal modes of the electromagnetic field, $Q_{smn}$ is the spherical wave coefficient and ${\vec{F}}_{smn}^{\left(3\right)}$ is the spherical wave basis function. In Equation (4), *k* is a wave number determined by the measurement frequency and *a* is the radius of the sphere with the smallest volume containing the entire OUT to be measured. *N* is the truncation number and is a variable that determines the minimum amount of data required for valid RCS prediction from the limited measurement data. Therefore, the number of m and *n* are determined to be N+2 and *N*, respectively.

In this case, the spherical wave basis function ${\vec{F}}_{smn}^{\left(3\right)}$, which takes the form of a vector, is calculated with both theta and phi directional components.

(5) $${\vec{F}}_{1mn}^{\left(3\right)}\left(r,\theta ,\phi \right)=\frac{1}{\sqrt{2\pi }}\frac{1}{\sqrt{n(n+1)}}{\left(\frac{-m}{\left|m\right|}\right)}^{m}\left[z_{n}^{\left(3\right)}\left(kr\right)\frac{im{\bar{P}}_{n}^{\left|m\right|}\left(\cos\theta \right)}{\sin\theta }e^{im\phi }\hat{\theta }-z_{n}^{\left(3\right)}\left(kr\right)\frac{d{\bar{P}}_{n}^{\left|m\right|}\left(\cos\theta \right)}{d\theta }e^{im\phi }\hat{\phi }\right]$$

(6) $$\begin{array}{cc} {\vec{F}}_{2mn}^{\left(3\right)}\left(r,\theta ,\phi \right)= & \frac{1}{\sqrt{2\pi }}\frac{1}{\sqrt{n(n+1)}}{\left(\frac{-m}{\left|m\right|}\right)}^{m}[\frac{n(n+1)}{kr}z_{n}^{\left(3\right)}\left(kr\right){\bar{P}}_{n}^{\left|m\right|}\left(\cos\theta \right)e^{im\phi }\hat{r}+\\ & \frac{1}{kr}\frac{d}{d(kr)}\left\{krz_{n}^{\left(3\right)}\left(kr\right)\right\}\frac{d{\bar{P}}_{n}^{\left|m\right|}\left(\cos\theta \right)}{d\theta }e^{im\phi }\hat{\theta }+\frac{1}{kr}\frac{d}{d(kr)}\left\{krz_{n}^{\left(3\right)}\left(kr\right)\right\}\frac{im{\bar{P}}_{n}^{\left|m\right|}\left(\cos\theta \right)}{\sin\theta }e^{im\phi }\hat{\phi }] \end{array}$$

(7) $$z_{n}^{\left(3\right)}\left(x\right)=j_{n}\left(x\right)+iy_{n}\left(x\right)$$

(8) $$j_{n}\left(x\right)={\left(-x\right)}^{n}{\left(\frac{1}{x}\frac{d}{dx}\right)}^{n}\frac{\sin x}{x}$$

(9) $$y_{n}\left(x\right)=-{\left(-x\right)}^{n}{\left(\frac{1}{x}\frac{d}{dx}\right)}^{n}\frac{\cos x}{x}$$

(10) $${\bar{P}}_{n}^{m}\left(x\right)={\left(-1\right)}^{m}\sqrt{\frac{\left(n+\frac{1}{2}\right)\left(n-m\right)!}{(n+m)!}}P_{n}^{m}\left(x\right)$$

(11) $$P_{n}^{m}\left(x\right)={\left(-1\right)}^{m}{\left(1-x^{2}\right)}^{m/2}\frac{d^{m}}{dx^{m}}\left(P_{n}\left(x\right)\right)$$

where $z_{n}^{\left(3\right)}\left(x\right)$ is the first kind spherical Hankel function consisting of the sum of the spherical Bessel function $j_{n}\left(x\right)$ and spherical Neumann function $y_{n}\left(x\right)$. ${\bar{P}}_{n}^{m}$ is the normalized associated Legendre polynomial, and $P_{n}^{m}$ is the original associated Legendre polynomial.

**Figure 3:**
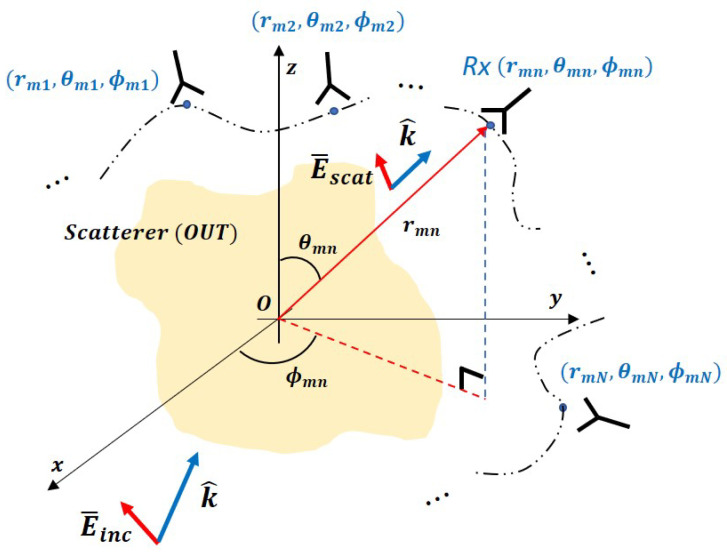
SWE field modeling at the measurement point for a scatterer.

As shown in Figure 3, if an electric field is expressed by SWE at an arbitrary near-field measurement point $\left(r_{mn},\theta_{mn},\phi_{mn}\right)$, then an N(N+2)-weighted sum is generated. This indicates that there are N(N+2) unknown coefficients, which can be derived by constructing a linear system of equations from the near-field measured sample values.

Based on Equations (4)–(11), Equation (3) can be expressed in the form of a matrix-vector product:

(12) $$U=CQ=\left[\begin{array}{cccc} F_{\theta ,s(-1)1}\left(r_{m1},\theta_{m1},\phi_{m1}\right) & F_{\theta ,s01}\left(r_{m1},\theta_{m1},\phi_{m1}\right) & \cdots & F_{\theta ,sNN}\left(r_{m1},\theta_{m1},\phi_{m1}\right)\\ F_{\phi ,s(-1)1}\left(r_{m1},\theta_{m1},\phi_{m1}\right) & F_{\phi ,s01}\left(r_{m1},\theta_{m1},\phi_{m1}\right) & \cdots & F_{\phi ,sNN}\left(r_{m1},\theta_{m1},\phi_{m1}\right)\\ F_{\theta ,s(-1)1}\left(r_{m2},\theta_{m2},\phi_{m2}\right) & F_{\theta ,s01}\left(r_{m2},\theta_{m2},\phi_{m2}\right) & \cdots & F_{\theta ,sNN}\left(r_{m2},\theta_{m2},\phi_{m2}\right)\\ \vdots & \vdots & \ddots & \vdots \\ F_{\theta ,s(-1)1}\left(r_{mn},\theta_{mn},\phi_{mn}\right) & F_{\theta ,s01}\left(r_{mn},\theta_{mn},\phi_{mn}\right) & \cdots & F_{\theta ,sNN}\left(r_{mn},\theta_{mn},\phi_{mn}\right)\\ F_{\phi ,s(-1)1}\left(r_{mn},\theta_{mn},\phi_{mn}\right) & F_{\phi ,s01}\left(r_{mn},\theta_{mn},\phi_{mn}\right) & \cdots & F_{\phi ,sNN}\left(r_{mn},\theta_{mn},\phi_{mn}\right) \end{array}\right]\left[\begin{array}{c} Q_{s(-1)1}\\ Q_{s01}\\ Q_{s11}\\ \vdots \\ \vdots \\ Q_{sNN} \end{array}\right]$$

(13) $$Q={\left(C^{H}C\right)}^{-1}C^{H}U$$

In Equations (12) and (13), *U* is a measured near-field sample vector that includes both θ and ϕ polarization, and *C* is a matrix that can be calculated from the coordinates of each measurement point. The subscripts θ and ϕ of the elements *F* constituting the matrix *C* represent the coefficients of the theta and phi components in Equations (5) and (6), respectively. *H* denotes the Hermitian operation of a matrix. From *U* and *C*, the unknown vector *Q* can be derived as shown in Equation (13). This process is the inverse problem illustrated in Figure 1b and represents the equivalent current source model for an OUT. The electric field in the far-field region is computed by substituting the $r_{m}$ value of each measurement coordinate with a significantly large value instead of using the *C* matrix calculated for the near-field measurement points.

(14) $$E_{far}=C_{far}Q=\left[\begin{array}{cccc} F_{\theta ,s(-1)1}\left(\infty ,\theta_{m1},\phi_{m1}\right) & F_{\theta ,s01}\left(\infty ,\theta_{m1},\phi_{m1}\right) & \cdots & F_{\theta ,sNN}\left(\infty ,\theta_{m1},\phi_{m1}\right)\\ F_{\phi ,s(-1)1}\left(\infty ,\theta_{m1},\phi_{m1}\right) & F_{\phi ,s01}\left(\infty ,\theta_{m1},\phi_{m1}\right) & \cdots & F_{\phi ,sNN}\left(\infty ,\theta_{m1},\phi_{m1}\right)\\ F_{\theta ,s(-1)1}\left(\infty ,\theta_{m2},\phi_{m2}\right) & F_{\theta ,s01}\left(\infty ,\theta_{m2},\phi_{m2}\right) & \cdots & F_{\theta ,sNN}\left(\infty ,\theta_{m2},\phi_{m2}\right)\\ \vdots & \vdots & \ddots & \vdots \\ F_{\theta ,s(-1)1}\left(\infty ,\theta_{mn},\phi_{mn}\right) & F_{\theta ,s01}\left(\infty ,\theta_{mn},\phi_{mn}\right) & \cdots & F_{\theta ,sNN}\left(\infty ,\theta_{mn},\phi_{mn}\right)\\ F_{\phi ,s(-1)1}\left(\infty ,\theta_{mn},\phi_{mn}\right) & F_{\phi ,s01}\left(\infty ,\theta_{mn},\phi_{mn}\right) & \cdots & F_{\phi ,sNN}\left(\infty ,\theta_{mn},\phi_{mn}\right) \end{array}\right]\left[\begin{array}{c} Q_{s(-1)1}\\ Q_{s01}\\ Q_{s11}\\ \vdots \\ \vdots \\ Q_{sNN} \end{array}\right]$$

The newly calculated $C_{far}$ matrix with r→∞ is multiplied by the solved vector Q. This process is the direct problem. By substituting the $E_{far}$ obtained in the same process as Equation (14) into $E_{scat}$ in Equation (1), we can predict the far-field RCS for each near-field measurement point. Each component *F* in the matrix $C_{far}$ can be computed for arbitrary measurement points that are not limited to specific canonical surfaces. This means that far-field RCS prediction using SWE-based NFFFT is possible if at least N(N+2) measurement samples are secured by the minimum truncation number *N*, as determined by Equation (4). In this study, in preparation for increases in the matrix size, the least squares QR-factorization (LSQR) method was utilized instead of a direct solver.

## 3. Numerical Results and Discussion

### 3.1. SWE-NFFFT Verification

**Figure 4:**
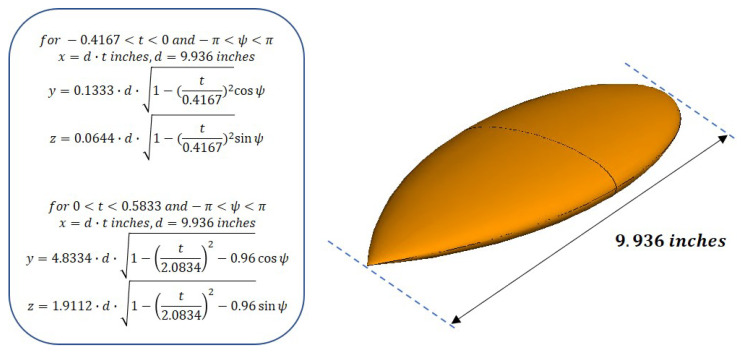
NASA almond-type computer aided design (CAD) model used for simulations.

This section describes the validation of the SWE-NFFFT algorithm proposed in Section 2. The far-field reference and near-field data at a specific distance are simulated using a three-dimensional full-wave electromagnetic solver FEKO based on method of moments (MoM) analysis. A perfect electrically conducting NASA almond-type OUT is considered. RCS measurement values have been well documented at specific frequencies for this type of object [24]. As shown in Figure 4, this object can be expressed as a closed surface formed by (x(t,ψ),y(t,ψ),z(t,ψ)) according to the positional variables t,ψ.

First, when the SWE-NFFFT procedure is utilized with electrical field data extracted from a distance (other than infinity) that is close to the far-field region, it is necessary to verify whether the OUT is correctly modeled with an equivalent current source. The incident waves are set as θ-polarized plane waves incident from a single direction of $\theta =90^{\circ }$ and $\phi =0^{\circ }$. The resulting near and far-field scattered wave data are all extracted in a bistatic format. The polarization data used for transformation, based on Equation (12), are θ and ϕ-pol.

**Figure 5:**
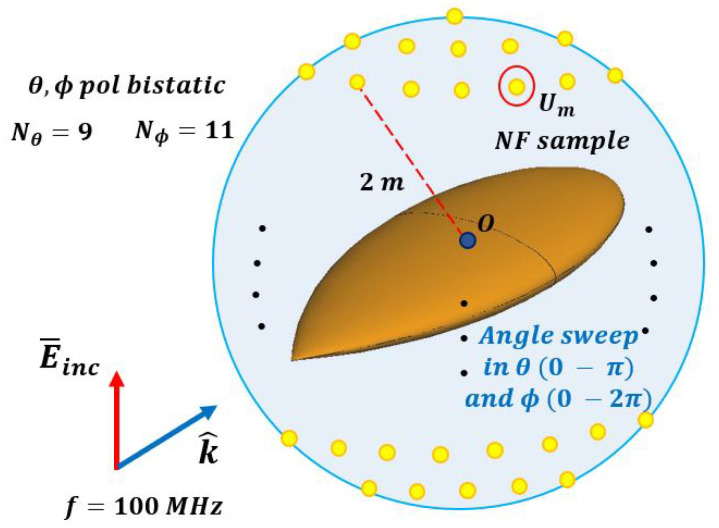
Simulation setup for near-field sample data from a spherical scanning surface.

**Figure 6:**
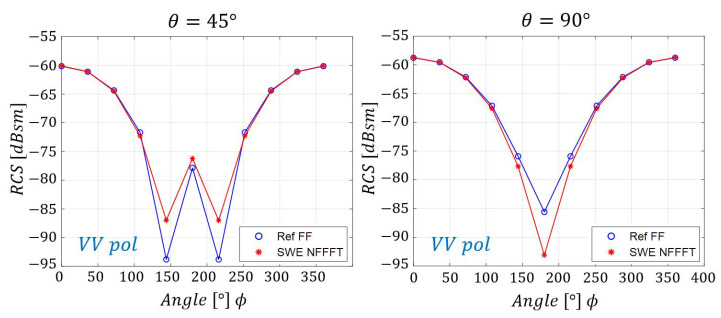
VV-pol SWE-NFFFT results for a 100 MHz incident wave on a $\theta =45^{\circ },90^{\circ }$ cut plane.

To verify the compatibility of SWE-NFFFT with a canonical surface, a spherical scan was performed with a radius of 2 m for the entire azimuth and elevation angle, as shown in Figure 5. The frequency of the incident wave was set to 100 MHz. Here, 99 near-field samples (N) were required for each polarization, as shown in Equation (4). For the ease of extraction of near-field data, the samples in the θ and ϕ directions were extracted at consistent intervals of $N_{\theta }=9$ and $N_{\phi }=11$, respectively. Under these simulation conditions, the size of the matrix *C* in Equation (12), which was required for SWE-NFFFT, was 198×198 and the size of the unknown vector *Q* was 198×1. Figure 6 presents the far-field RCS prediction results based on the vector *Q* obtained from SWE-NFFFT. Among the 99 far-field RCS data points predicted for all azimuth angles, the vertical transmit and vertical receive polarization (VV-pol) RCS results are displayed for $\theta =45^{\circ }$ and $\theta =90^{\circ }$ cut planes. The solid line composed of blue circles represents the reference far-field RCS, whereas the solid line composed of red stars represents the prediction results when using the SWE-NFFFT method. It is clear that the SWE-NFFFT-based RCS values show excellent agreement, except for the extremely small RCS values, which would be negligible a in practical scenario.

We then considered a frequency of 300 MHz, and all other simulation conditions were kept the same. Based on this increased operating frequency, the number of near-field samples per polarization was 323. The samples in the θ and ϕ directions were extracted at consistent intervals of $N_{\theta }=17$ and $N_{\phi }=19$, respectively.

**Figure 7:**
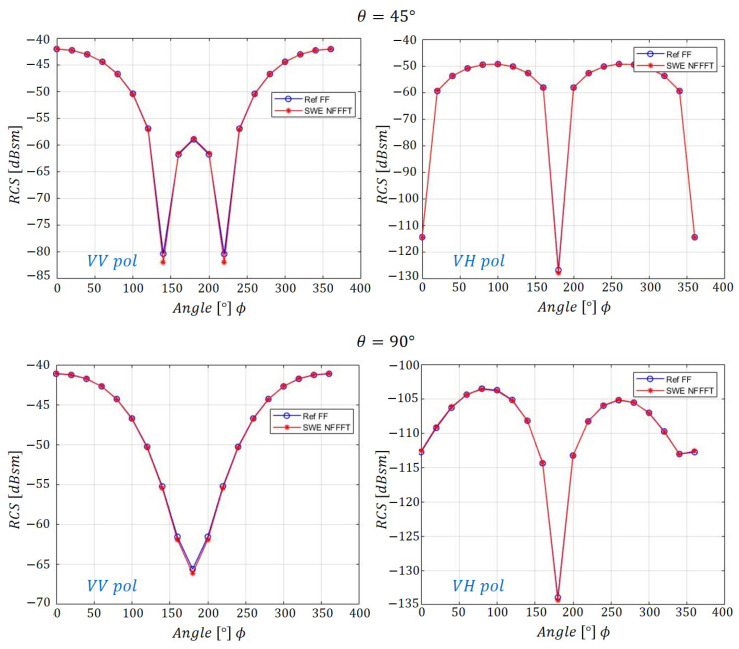
SWE-NFFFT results for a 300 MHz incident wave on a $\theta =45^{\circ },90^{\circ }$ cut plane.

Figure 7 presents the RCS data for various cases with $\theta =45^{\circ }$ and $\theta =90^{\circ }$ cut planes for VV and VH(-horizontal receive)-pol. Based on the verification processes above, it is confirmed that applying the SWE-NFFFT method is reasonable in the case of spherical scanning at a distance in the far-field region.

We then analyzed the applicability of the SWE-NFFFT method to various scanning surfaces. We also examined tge partial surface scans and compared the results to electric field integral equation-based NFFFT, which is dependent on a canonical scanning surface and requires scanning for all-round surfaces (e.g., spheres and cylinders). Additionally, to verify the validity of RCS prediction in the near-field region, field samples were extracted at a shorter distance compared to the previous simulation.

**Figure 8:**
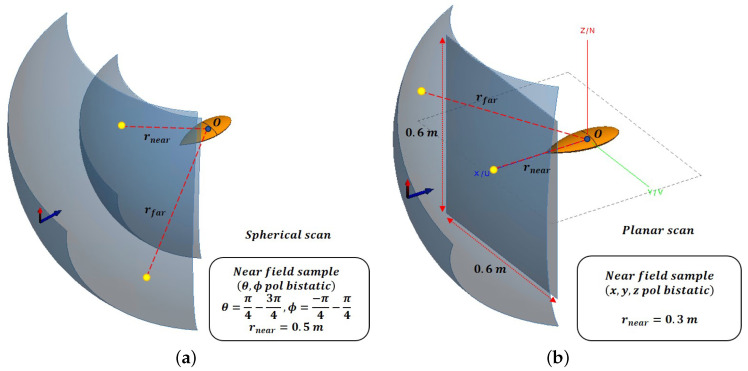
Simulation setup for near-field sample data from two canonical scanning surfaces. (**a**) Spherical near-field scan. (**b**) Planar near-field scan.

As shown in Figure 8, verification was performed for two cases: a scan of a spherical surface and a square plane surface containing the same angular region ($\theta =\frac{\pi }{4}∼\frac{3\pi }{4}$, $\phi =\frac{-\pi }{4}∼\frac{\pi }{4}$) with the same frequency of 300 MHz. In the spherical scan, all near-field sample points were extracted at a radius of 0.5 m, whereas in the planar scan, the nearest sample point was located at a distance of 0.3 m on a square plane with a size of 0.6×0.6 m. To compare the far-field RCS results at identical sample points for the two scanning methods, we considered values of $N_{\theta }=19$ and $N_{\phi }=21$, which were greater than the minimum truncation number required to have the same θ and ϕ values. Based on Equations (5), (6) and (12), SWE-NFFFT was calculated for matrix *C* under θ polarization and ϕ polarization in the transformation process. In the case of the planar scan, the x,y,z polarization components were extracted and then transformed using Equations (15)–(18);

(15) $$X\hat{\theta }=A\hat{x}+B\hat{y}+C\hat{z}$$

(16) $$Y\hat{\phi }=D\hat{x}+E\hat{y}$$

(17) X=Acosθcosϕ+Bcosθsinϕ−Csinθ

(18) Y=−Dsinθ+Esinϕ

**Figure 9:**
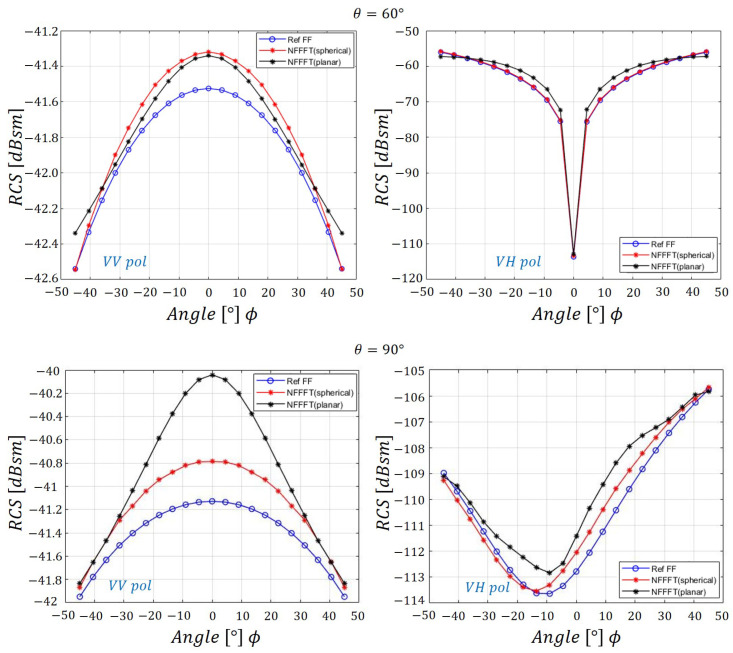
Comparison of SWE-NFFFT results according to the scanning method ($\theta =60^{\circ },90^{\circ }$ cut plane).

Figure 9 presents the RCS data from the two different scanning methods and the reference values, which show excellent agreement.

The VV-pol as well as VH-pol RCS results at the cut planes of $\theta =60^{\circ }$ and $\theta =90^{\circ }$ are presented in Figure 9, revealing a maximum error of 2 dB, which is almost negligible. The solid line composed of blue circles represents the reference far-field RCS, and the solid line composed of red stars represents the spherical scan NFFFT; the black solid line represents the planar scan NFFFT results. In the $\theta =90^{\circ }$ cut plane, for VV-pol, a maximum error of 1 dB occurs at $\phi =0^{\circ }$. In the case of the VH-pol, the maximum error is approximately 2 dB around $\phi =10^{\circ }$. In the case of a planar scan, because each near-field sample point has a different distance, larger errors occur compared to the spherical scan, but reasonable NFFFT results are obtained in general.

### 3.2. Far-Field RCS Prediction on Arbitrary Scanning Surfaces

In this section, the proposed SWE-NFFFT algorithm is applied to predict the far-field RCS for an arbitrary scanning surface on a more realistic OUT model.

**Figure 10:**
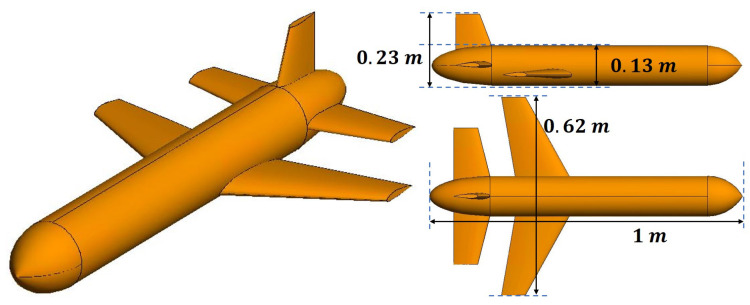
Missile CAD model used for simulation.

Compared to the streamlined NASA almond, the OUT model used in this simulation has the shape of a missile with a length of 1 m and width of 0.62 m, as shown in Figure 10.

The direction and parameters of the incident wave were the same as those mentioned in the previous section and the frequency was increased to 1 GHz. By setting N=34, which is greater than the minimum truncation number determined by Equation (4), the far-field RCS can be calculated from the matrix *C* (2448×2448). When considering the size of the missile-shaped OUT and the wavelength, the minimum far-field region is 7 m. The near-field measurement data were extracted within a range of 3–5 m, and θ and the distance vector *r* were set to different random values to ensure that the scanning surface did not correspond to a specific canonical surface. A total of 2448 sample data points were extracted from 12 surfaces (102 points at $1^{\circ }$ intervals, with 1224 samples for each polarization).

**Figure 11:**
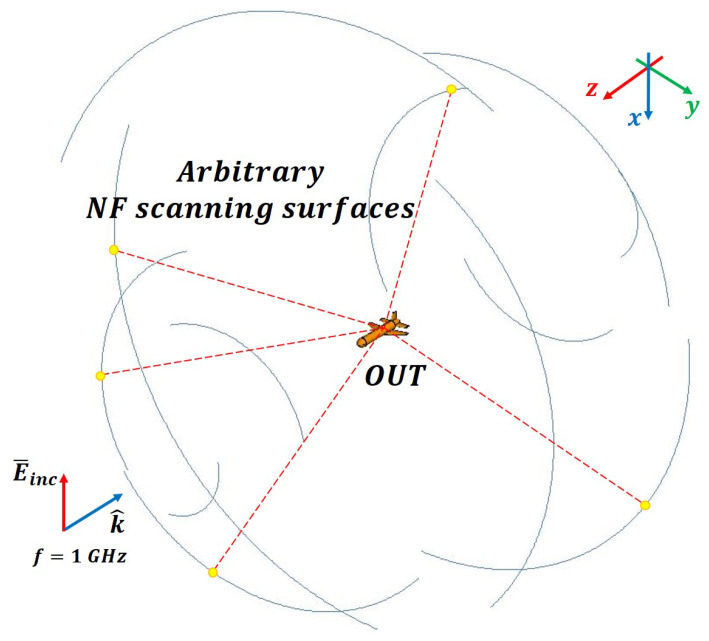
Simulation setup for arbitrary scanning surfaces.

**Figure 12:**
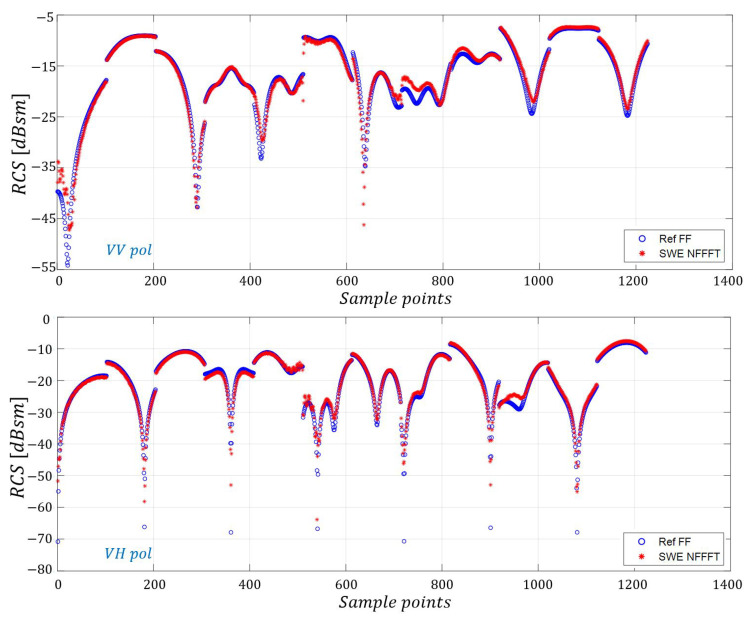
Comparisons of SWE-NFFFT results for all near-field sample points.

**Figure 13:**
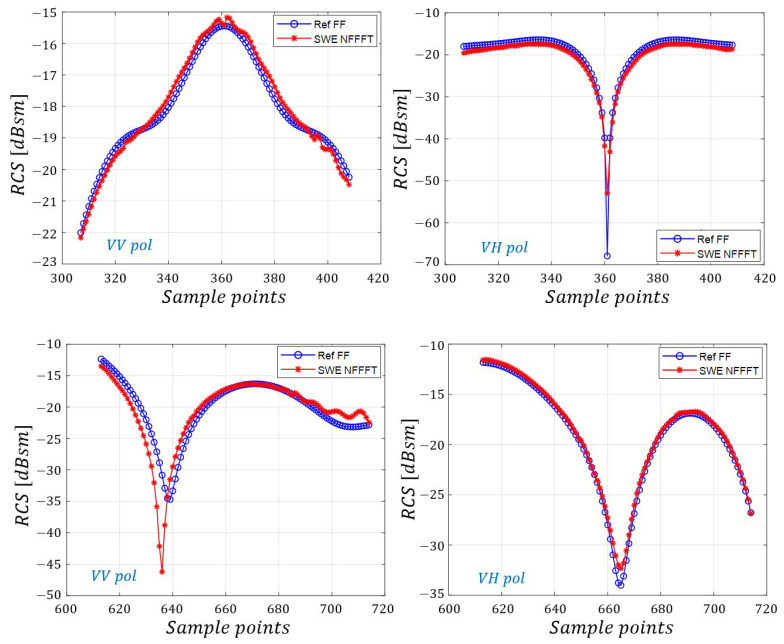
Partial plot of SWE-NFFFT results for all near-field sample points (306∼407, 612∼713).

The SWE-NFFFT-based RCS data are compared with the reference data for 12 different scanning surfaces under VV and VH-pol in Figure 11 and Figure 12, respectively, revealing excellent agreement. Figure 13 presents more detailed data.

It is clear that the proposed SWE-NFFFT method enables the prediction of high-accuracy far-field RCS data with arbitrary scanning surfaces for general OUTs.

## 4. Conclusions

In this paper, a SWE-NFFFT method was proposed for arbitrary measurement points that do not belong to canonical surfaces, and it was proven that the proposed method is applicable to near-field RCS measurement systems. Therefore, any arbitrary near-field data can be utilized to derive far-field RCS data.

First, a system of linear equations is generated from the minimum near-field sample data required for far-field RCS prediction. When the distance and angular coordinates of each measurement point are specified, spherical wave functions that are orthogonal to each other can be calculated. The coefficients multiplied by weights for each function can be derived as determinants by assuming unknown vectors. In the system of linear equations, an unknown vector is calculated as an inverse problem using the LSQR method. It is then modeled as an equivalent current source, and the far-field electric field is derived from the modeled source as a direct problem. Finally, the RCS can be predicted.

In this study, the compatibility of a conventional canonical scanning surface was verified for different frequencies and measurement distances using a NASA almond-type OUT. Next, the proposed SWE-NFFFT was validated on an arbitrary scanning surface for a more general shape of an OUT in the GHz frequency region. Finally, it was verified that RCS prediction can be applied to any arbitrary scanning surface. Therefore, it has been confirmed that RCS prediction using the proposed SWE-based NFFFT algorithm is feasible. In particular, in the final verification process for a missile-shaped OUT, the overall RCS tendency showed high accuracy. It was confirmed that about a 3 dB error occurred at several points, which was about 10 dB at the null point, which would be negligible in a practical scenario.

In future work, we plan to extend the NFFFT method for non-uniform sampling, which extracts near-field sample data more densely from measurement points with large error.
